# Association of Age-Related Hearing Impairment With Physical Functioning Among Community-Dwelling Older Adults in the US

**DOI:** 10.1001/jamanetworkopen.2021.13742

**Published:** 2021-06-25

**Authors:** Pablo Martinez-Amezcua, Danielle Powell, Pei-Lun Kuo, Nicholas S. Reed, Kevin J. Sullivan, Priya Palta, Moyses Szklo, Richey Sharrett, Jennifer A. Schrack, Frank R. Lin, Jennifer A. Deal

**Affiliations:** 1 Department of Epidemiology, Johns Hopkins Bloomberg School of Public Health, Baltimore, Maryland; 2 Center on Aging and Health, Johns Hopkins University, Baltimore, Maryland; 3 Cochlear Center for Hearing and Public Health, Baltimore, Maryland; 4 Intramural Research Program, National Institute on Aging, Baltimore, Maryland; 5 Department of Medicine, University of Mississippi, Medical Center, Jackson; 6 Department of Medicine, Division of General Medicine, Columbia University Irving Medical Center, New York, New York; 7 Department of Epidemiology, Joseph P. Mailman School of Public Health, Columbia University Irving Medical Center, New York, New York; 8 Welch Center for Prevention, Epidemiology and Clinical Research, Johns Hopkins Bloomberg School of Public Health, Baltimore, Maryland

## Abstract

**Importance:**

Hearing impairment, a common treatable condition, may contribute to poorer physical function with aging.

**Objective:**

To assess whether hearing impairment is associated with poorer physical function, reduced walking endurance, and faster decline in physical function.

**Design, Setting, and Participants:**

In this cohort study, cross-sectional and longitudinal analyses were performed using data from the 2011 to 2019 period of the Atherosclerosis Risk in Communities study, a population-based study of community-dwelling adults at 4 sites in the US.

**Exposures:**

Hearing thresholds (per 10 dB) assessed with pure tone audiometry and categorized as normal hearing or mild, moderate, or severe hearing impairment.

**Main Outcomes and Measures:**

Physical function was assessed using the short physical performance battery (SPPB), with composite scores ranging from 0 to 12. A composite score of 6 or less and a score for each component (balance, gait speed, and chair stands) of 2 or less indicated poor performance. Walking endurance was assessed using a 2-minute fast-paced walk test. Tobit regression models adjusted for sociodemographic factors and medical history were used to calculate the mean differences in SPPB composite scores; logistic regression models, to estimate the odds ratios (ORs) of low SPPB composite and component scores; and linear mixed-effects models, to estimate the mean rate of change in SPPB composite scores over time.

**Results:**

Of the 2956 participants (mean [SD] age, 79 [4.6] years) who attended study visit 6 between 2016 and 2017, 1722 (58.3%) were women, and 2356 (79.7%) were White. As determined by pure tone audiometry, 973 (33%) participants had normal hearing, 1170 (40%) had mild hearing impairment, 692 (23%) had moderate hearing impairment, and 121 (4%) had severe hearing impairment. In the Tobit regression model, severe hearing impairment was associated with a lower mean SPPB score (β, –0.82; 95% CI, –0.34 to –1.30) compared with normal hearing. In fully adjusted logistic regression models, hearing impairment was associated with higher odds of low physical performance scores (severe impairment vs normal hearing: OR for composite physical performance, 2.51 [95% CI, 1.47-4.27]; OR for balance, 2.58 [95% CI, 1.62-4.12]; OR for gait speed, 2.11 [95% CI, 1.03-4.33]). Over time (2 to 3 visits; maximum, 8.9 years), participants with hearing impairment had faster declines in SPPB compared with those with normal hearing (moderate hearing impairment × time interaction, –0.34 [–0.52 to –0.16]). In adjusted models for walking endurance, participants with moderate or severe hearing impairment walked a mean distance of –2.81 m (95% CI, –5.45 to –0.17 m) and –5.31 m (95% CI, –10.20 to –0.36 m) than those with normal hearing, respectively, during the 2-minute walk test.

**Conclusions and Relevance:**

In this cohort study, hearing impairment was associated with poorer performance, faster decline in physical function, and reduced walking endurance. The results of the longitudinal analysis suggest that hearing impairment may be associated with poorer physical function with aging. Whether management of hearing impairment could delay decline in physical function requires further investigation.

## Key Points

**Question:**

Is hearing impairment associated with poorer physical function, reduced walking endurance, and faster decline in physical function?

**Findings:**

In this population-based cohort study of 2956 older adults in the US, participants with hearing impairment had significantly poorer physical function (particularly balance), worse walking endurance (over a 2-minute walk), and faster declines in physical function over time compared with those with normal hearing.

**Meaning:**

This study’s findings suggest that because hearing impairment is a prevalent but treatable condition, it may be a target for interventions to slow the decline of physical function associated with aging.

## Teaser

This cohort study assesses the association between hearing impairment and decline in physical function in terms of balance and walking endurance in a group of community-dwelling older adults in the US.

## Introduction

Physical functioning is necessary for independent living and tends to decline with age.^1,2,3^ Hearing impairment, which affects approximately two-thirds of adults older than 70 years,^4^ is a risk factor for various adverse outcomes.^5,6,7^ Hearing impairment may also adversely affect physical functioning through reduced perception of auditory input that contributes to walking and balance.^8^ However, research characterizing the association between hearing impairment and objective physical function and walking endurance measures is limited.

Associations between self-reported hearing impairment and poorer physical function have been reported previously.^9,10^ However, self-reported hearing impairment is prone to measurement error and has been shown to underestimate associations with objective measures of function.^11^ Although studies with audiometrically assessed hearing, the criterion-standard clinical measure, have revealed associations with slower gait and poorer physical function,^12,13,14^ these studies did not assess associations with physical function components separately. Moreover, studies of the association between hearing impairment and walking endurance—the ability to walk longer distances, an early factor associated with disability^15^—are scarce and have inconsistent findings.^10,16^

Therefore, we investigated the association of hearing impairment with physical function and walking endurance in a cohort of community-dwelling older adults in the US. We hypothesized that participants with hearing impairment would have poorer concurrent physical function and walking endurance and a faster decline in physical function over approximately 8 years compared with participants with normal hearing.

## Methods

### Study Population

For this cohort study, we used a population from the Atherosclerosis Risk in Communities (ARIC) study,^17^ an ongoing study of community-dwelling adults that enrolled 15 792 participants between 1987 and 1989 at 4 sites in the US (Washington County, Maryland, Forsyth County, North Carolina, Minneapolis, Minnesota, and Jackson, Mississippi). Hearing was assessed during an in-person examination at visit 6 (2016-2017). We used physical function data collected during clinic visits 5 (2011-2013) through 7 (2018-2019). The institutional review board at each site approved the ARIC study, and all participants provided written informed consent. Consent for the ARIC study covered use of the data for all analyses approved by the study publications committee, and data were deidentified. The present study was approved by the institutional review board of the Johns Hopkins Bloomberg School of Public Health. This study followed the Strengthening the Reporting of Observational Studies in Epidemiology (STROBE) reporting guidelines for cohort studies.^18^

Among 4003 participants who attended visit 6, 3395 had complete evaluation of physical function. After exclusion of 103 participants with missing information for the hearing assessment and 336 participants with missing information for the covariates of interest (race [n = 17], occupational noise [n = 39], smoking [n = 199], body mass index [n = 26], educational level [n = 6], multimorbidity [n = 5], and gait speed [n = 58]), the population for cross-sectional physical function analyses included 2956 participants. Longitudinal data (up to 8.9 years from visit 5 to visit 6; 2-3 observations) were available for 2869 participants (7901 observations; mean [SD] follow-up time from visit 5 to visit 6, 3.6 [2.8] years). Walking endurance of 2961 participants was evaluated at visit 6; 2532 had complete data (39 did not complete the endurance test, 64 had missing exposure, and 362 had missing information for covariates) and were included in the analytic population for walking endurance analysis.

### Exposure

Pure tone audiometry, assessed at ARIC visit 6 (2016-2017), was completed in a soundproof booth using insert earphones (EARTone 3a; 3M) and an Interacoustics AD629 or Equinox audiometer (Interacoustics A/S). Measurement of air conduction was completed at standard octaves from 500 to 8000 Hz. For each frequency, the hearing threshold was recorded in decibels hearing level (dB HL). We calculated a 4-frequency (0.5, 1, 2, and 4 kHz) pure tone average for each ear and modeled a continuous measure of hearing (better-hearing ear pure tone average [BPTA]) scaled to 10 dB HL, with higher BPTA indicating worse hearing function. Hearing was also categorized according to World Health Organization standards as normal hearing (BPTA≤25 dB HL), mild hearing impairment (BPTA of 26-40 dB HL), moderate hearing impairment (BPTA of 41-60 dB HL), or severe hearing impairment (BPTA>60 dB HL).^19^

### Outcomes

The Short Physical Performance Battery (SPPB) was used to assess physical function, and a fast-paced 2-minute walk (TMW) test was used to measure walking endurance. The SPPB was measured at visits 5, 6, and 7 and included 3 components: balance, gait speed, and chair stands. Each component was scored on a scale of 0 to 4 points, with higher scores indicating better physical function (eTable 1 in the Supplement). The SPPB has an established scoring system, which we used to derive cutoffs for each test from the performance distribution.^20^ The composite SPPB score (range, 0-12) was the sum of the 3 scores. We examined the composite SPPB score continuously and used a binary threshold for poor physical performance (SPPB composite score ≤6).^1,20^ For each SPPB component, we also used a binary threshold (SPPB component score ≤2) to indicate poor performance, consistent with prior work (eTable 1 in the Supplement).^21^

The balance test consisted of holding 3 standing positions for 10 seconds each: side-by-side (easiest), semi-tandem, and full-tandem (hardest)^20^; participants progressed to a more difficult position if they succeeded in 1 of 2 trials. Each participant’s usual pace gait speed was measured twice over a 4-m walk (walking aids permitted). We calculated gait speed (in meters per second) using the trial with the faster result. For the chair stand test, participants stood up from a chair and sat back down with arms crossed 5 times as quickly as possible. Gait speed and time to complete the chair stands were also examined continuously.

The TMW test was administered on a 15.24-m (50-ft) course at visit 6 only. Participants were instructed to walk as fast as they could for 2 minutes. The distance covered was recorded in meters. Participants unable to complete the SPPB 4-m walk test unaided were excluded from the TMW test, leaving 2535 participants for this analysis.

### Additional Independent Variables

Age; sex; educational level (less than high school, high school, or high school or higher); race–study site, owing to the correlation between race and site in the ARIC study^17^ (White participants from Minneapolis, Washington County, and Forsyth County and Black participants from Forsyth County and Jackson); body mass index; occupational noise exposure (very loud sounds for >10 hours/wk in the workplace); and smoking status (never, former, or current) were assessed at visit 6. Using data from the annual follow-up interviews, we created a multimorbidity index to control for the presence of chronic conditions. This index was modeled after the Charlson-Deyo Comorbidity Index and included myocardial infarction, stroke, intermittent claudication, heart failure, chronic obstructive pulmonary disease (emphysema or bronchitis), chronic kidney disease, diabetes, and cancer. Stroke and cancer were assigned double weight (counted as 2). No data were available regarding peptic ulcer disease, AIDS or HIV infection, liver disease, or rheumatologic disease. Dementia was not included in the index because we considered it to be a mediator and not a confounder in the association between hearing and physical function. In sensitivity analyses, we tested whether including dementia in the index had any effect on our results. All covariates were assessed at visit 6 and treated as fixed variables.

### Statistical Analysis

We evaluated the differences in demographic characteristics and medical conditions across hearing categories using χ^2^ and analysis of variance tests as appropriate. We assessed the cross-sectional associations (at study visit 6) between continuous hearing, by 10-dB HL increments, and physical function and walking endurance using 2 models. Model 1 was adjusted for age, sex, and race–study center, and model 2 was adjusted for those variables as well as educational level, body mass index, occupational noise, smoking status, and the multimorbidity index.

Because of the ceiling effect of the SPPB (approximately 50% of participants had a score ≥10), we used Tobit regression models to calculate the mean differences in SPPB composite scores. We used logistic regression analysis to estimate the odds ratios (ORs) of low SPPB composite scores (≤6) and low scores for each SPPB component (≤2). We used linear regression analysis to assess differences in gait speed and time to complete chair stands as continuous measures.

We also used linear regression analysis to assess the cross-sectional (visit 6) association between hearing and walking endurance (distance walked during the TMW test). We performed all analyses using Stata, version 15 (StataCorp LLC). Two-sided *P* < .05 was considered statistically significant.

For longitudinal analyses (a description of the longitudinal design is given in the eFigure in the Supplement), we used linear mixed-effects models with an unstructured covariance matrix to estimate the mean rate of change in SPPB composite scores over time (measured in years from visit 5 [2011-2013] to visit 7 [2018-2019]) by hearing exposure. Because hearing was measured at visit 6 (2016-2017), we included a 2-piece linear spline term for time, with a knot 5 years after visit 5 (approximate mean time between visits 5 and 6) to allow the rate of decline in SPPB scores to vary between visits. We allowed for between-person heterogeneity in SPPB scores with random effects for intercept and slopes (before and after year 5). We included interaction terms between hearing and both time coefficients (before and after year 5) to estimate the mean differences in the rate of change in physical function across hearing categories in each period. We evaluated the assumptions of our models with residual plots and present fully adjusted longitudinal models. In a secondary analysis, among 811 participants with moderate or severe hearing impairment, we assessed whether there were differences in physical function and walking endurance by hearing aid use (self-reported as yes or no) using fully adjusted models.

For sensitivity analysis, using ordinal logistic regressions, we estimated the OR of having the next-lower SPPB score (the scores were reversed to compute OR) for all hearing groups. Also, among 2520 participants, we examined the association between hearing (per 10-dB HL increments and across hearing categories) and grip strength (a measure of the forearm’s maximal strength) using linear regressions adjusted for covariates in model 2. In addition, we built an alternative multimorbidity index including dementia and used it for adjustment in model 2 in lieu of the index without dementia.

## Results

A total of 2956 participants attended study visit 6 (Table 1). The mean (SD) age was 79 (4.6) years (range, 71-94 years); 1722 participants (58.3%) were women, and 2356 (79.7%) were White. Overall, 973 participants (33%) had normal hearing, 1170 (40%) had mild hearing impairment, 692 (23%) had moderate hearing impairment, and 121 (4%) had severe hearing impairment. Compared with participants with normal hearing, those with hearing impairment were more likely to be older, to be men, to be White individuals, to have a lower educational level, and to have more chronic conditions.

**Table 1.** Sociodemographic Characteristics, History of Chronic Conditions, Physical Function, and Walking Endurance by Hearing Status at Visit 6 in the ARIC Study^a^

Characteristic	Participants^b^				*P* value^c^
	Normal hearing (n = 973)	Mild HI (n = 1170)	Moderate HI (n = 692)	Severe HI (n = 121)	
Age, mean (SD), y	77.4 (3.7)	79.1 (4.4)	81.1 (4.8)	81.9 (5.3)	<.001
Sex					
Men	273 (28.1)	500 (42.7)	380 (54.9)	81 (66.9)	<.001
Women	700 (71.9)	670 (57.3)	312 (45.1)	40 (33.1)	
White race	659 (67.7)	960 (82.1)	622 (89.9)	115 (95.0)	<.001
BMI, mean (SD)	28.4 (5.4)	28.4 (5.4)	27.9 (5.0)	28.0 (4.8)	.25
Multimorbidity index, No. of conditions					
0	495 (50.9)	595 (50.9)	322 (46.5)	49 (40.5)	<.001
1-2	383 (39.4)	449 (38.4)	248 (35.8)	46 (38.0)	
≥3	95 (9.8)	126 (10.8)	122 (17.6)	26 (21.5)	
Educational level					
Less than high school	87 (8.9)	121 (10.3)	82 (11.9)	26 (21.5)	<.001
High school or equivalent	374 (38.4)	474 (40.5)	324 (46.8)	51 (42.2)	
More than high school	512 (52.2)	575 (49.2)	286 (41.3)	44 (36.4)	
Occupational noise exposure	159 (16.3)	275 (23.5)	225 (32.5)	53 (43.8)	<.001
Smoking status					
Never	413 (42.3)	437 (37.2)	279 (40.0)	41 (33.9)	.07
Former	487 (49.9)	664 (56.6)	367 (52.6)	69 (57.0)	
Current	76 (7.8)	75 (6.4)	52 (7.5)	11 (9.1)	
SPPB composite score, median (IQR)	10 (8-11)	10 (8-11)	9 (7-11)	9 (6-11)	<.001
Low SPPB composite score	131 (13.5)	206 (17.6)	147 (21.2)	36 (29.8)	<.001
Low SPPB balance score	192 (19.3)	336 (28.7)	253 (36.6)	55 (45.5)	<.001
Low SPPB gait speed score	66 (6.8)	120 (10.3)	76 (11.0)	14 (11.6)	.009
Low SPPB chair stand score	538 (55.3)	666 (56.9)	418 (60.4)	76 (62.8)	.12
Gait speed, mean (SD), m/s	0.96 (0.21)	0.94 (0.22)	0.91 (0.22)	0.89 (0.21)	<.001
Time to complete chair stands, mean (SD), s	14.4 (4.3)	14.6 (4.9)	14.8 (4.7)	14.6 (4.0)	.34
TMW, mean (SD), m	138.6 (27.2)	138.3 (29.1)	136.1 (8.7)	132.9 (28.5)	.11
BPTA, mean (SD), dB HL	18.9 (4.5)	32.6 (4.3)	48.6 (5.3)	69.4 (8.1)	<.001

Abbreviations: ARIC, Atherosclerosis Risk in Communities; BMI, body mass index (calculated as weight in kilograms divided by height in meters squared); BPTA, better-hearing ear pure tone average; HI, hearing impairment; IQR, interquartile range; SPPB, Short Physical Performance Battery; TMW, 2-minute walk.

^a^ Visit 6 occurred in 2016 or 2017.

^b^ Data are presented as number (percentage) of participants unless otherwise indicated.

^c^ *P* values were determined using χ^2^ for categorical variables and analysis of variance for continuous variables.

In a descriptive analysis (Table 1), compared with participants with normal hearing, greater proportions of participants with hearing impairment had low SPPB composite scores (normal, 131 [13.5%]; mild, 206 of 1170 [17.6%]; moderate, 147 of 692 [21.2%]; severe, 36 of 121 [29.8%]; *P* < .001), low balance scores (normal, 192 [19.3%]; mild, 336 of 1170 [28.7%]; moderate, 253 of 692 [36.6%]; severe, 55 of 121 [45.5%]; *P* < .001), and low gait speed scores (normal, 66 [6.8%]; mild, 120 of 1170 [10.3%]; moderate, 76 of 692 [11.0%]; severe, 14 of 121 [11.6%]; *P* = .01). The proportions of participants with a low chair stand score (normal, 538 of 973 [55.3%]; mild, 666 of 1170 [56.9%]; moderate, 418 of 692 [60.4%]; severe, 76 of 121 [62.8%]; *P* = .12) and the mean (SD) distance covered during the TMW test (normal, 138.6 [27.2] m; mild, 138.3 [29.1] m; moderate, 136.1 [8.7] m; severe, 132.9 [28.5] m; *P* = .11) were not significantly different across hearing groups.

### Cross-Sectional Analysis Findings

#### Physical Function

The differences in continuous measures of SPPB tests (composite score, gait speed, and time to complete chair stands) at visit 6, per 10-dB HL increase, and across hearing categories (in which normal hearing was the reference group) are presented in Table 2. Each 10-dB HL increase was associated with lower SPPB scores and slower gait speed. For example, per 10-dB HL increase, mean SPPB score was −0.17 points (95% CI, −0.24 to −0.10 points), and mean gait speed was −0.01 m/s (95% CI, −0.02 to −0.01 m/s). Similarly, for our binary outcomes, per 10-dB HL increase, the OR for a low SPPB composite score was 1.18 (95% CI, 1.09-1.29), and the ORs for low SPPB components scores were 1.21 (95% CI, 1.13-1.30) for balance, 1.18 (95% CI, 1.06-1.31) for gait speed, and 1.10 (95% CI, 1.03-1.17) for chair stands.

**Table 2.** Cross-Sectional Analysis of Associations Between BPTA and Hearing Categories and Continuous Measures of Physical Function and Walking Endurance at Visit 6 in the ARIC Study^a^

Outcome	β (95% CI)	
	Model 1^b^	Model 2^c^
SPPB composite score (n = 2956)^d^		
BPTA, per 10-dB HL decrease	−0.24 (−0.32 to −0.17)^e^	−0.17 (−0.24 to −0.10)^e^
Mild HI vs normal hearing	−0.44 (−0.67 to −0.21)^e^	−0.33 (−0.55 to −0.12)^e^
Moderate HI vs normal hearing	−0.71 (−0.98 to −0.43)^e^	−0.50 (−0.76 to −0.24)^e^
Severe HI vs normal hearing	−1.18 (−1.69 to −0.67)^e^	−0.82 (−1.30 to −0.34)^e^
Gait speed (n = 2956)		
BPTA, per 10-dB HL decrease	−0.02 (−0.02 to −0.01)^e^	−0.01 (−0.02 to −0.01)^e^
Mild HI vs normal hearing	−0.04 (−0.06 to −0.02)^e^	−0.03 (−0.05 to −0.02)^e^
Moderate HI vs normal hearing	−0.06 (−0.08 to −0.04)^e^	−0.04 (−0.06 to −0.02)^e^
Severe HI vs normal hearing	−0.08 (−0.12 to −0.04)^e^	−0.05 (−0.09 to −0.02)^e^
Time to complete 5 chair stands (n = 2535)		
BPTA, per 10-dB HL decrease	0.20 (0.07 to 0.34)^e^	0.12 (−0.01 to 0.26)
Mild HI vs normal hearing	0.39 (−0.03 to 0.80)	0.30 (−0.11 to 0.70)
Moderate HI vs normal hearing	0.70 (0.20 to 1.21)^e^	0.47 (−0.02 to 0.97)
Severe HI vs normal hearing	0.56 (−0.40 to 1.53)	0.21 (−0.73 to 1.16)
Distance walked during the TMW test (n = 2532)		
BPTA, per 10-dB HL decrease	−1.62 (−2.39 to −0.85)^e^	−0.98 (−1.69 to −0.26)^e^
Mild HI vs normal hearing	−2.87 (−5.21 to −0.53)^e^	−2.10 (−4.24 to 0.04)
Moderate HI vs normal hearing	−4.62 (−7.48 to −1.76)^e^	−2.81 (−5.45 to −0.17)^e^
Severe HI vs normal hearing	−7.96 (−13.3 to −2.58)^e^	−5.31 (−10.2 to −0.36)^e^

Abbreviations: ARIC, Atherosclerosis Risk in Communities; BPTA, better-hearing ear pure tone average; HI, hearing impairment; HL, hearing level; SPPB, Short Physical Performance Battery; TMW, 2-minute walk.

^a^ Visit 6 occurred in 2016 or 2017.

^b^ Model 1 was adjusted for age, sex, and race–center site.

^c^ Model 2 was adjusted for age, sex, race–center site, educational level, body mass index, occupational noise exposure, smoking status, and multimorbidity index.

^d^ The balance component of the SPPB was omitted; we used only a binary balance score.

^e^ Statistically significant.

**Figure 1.**
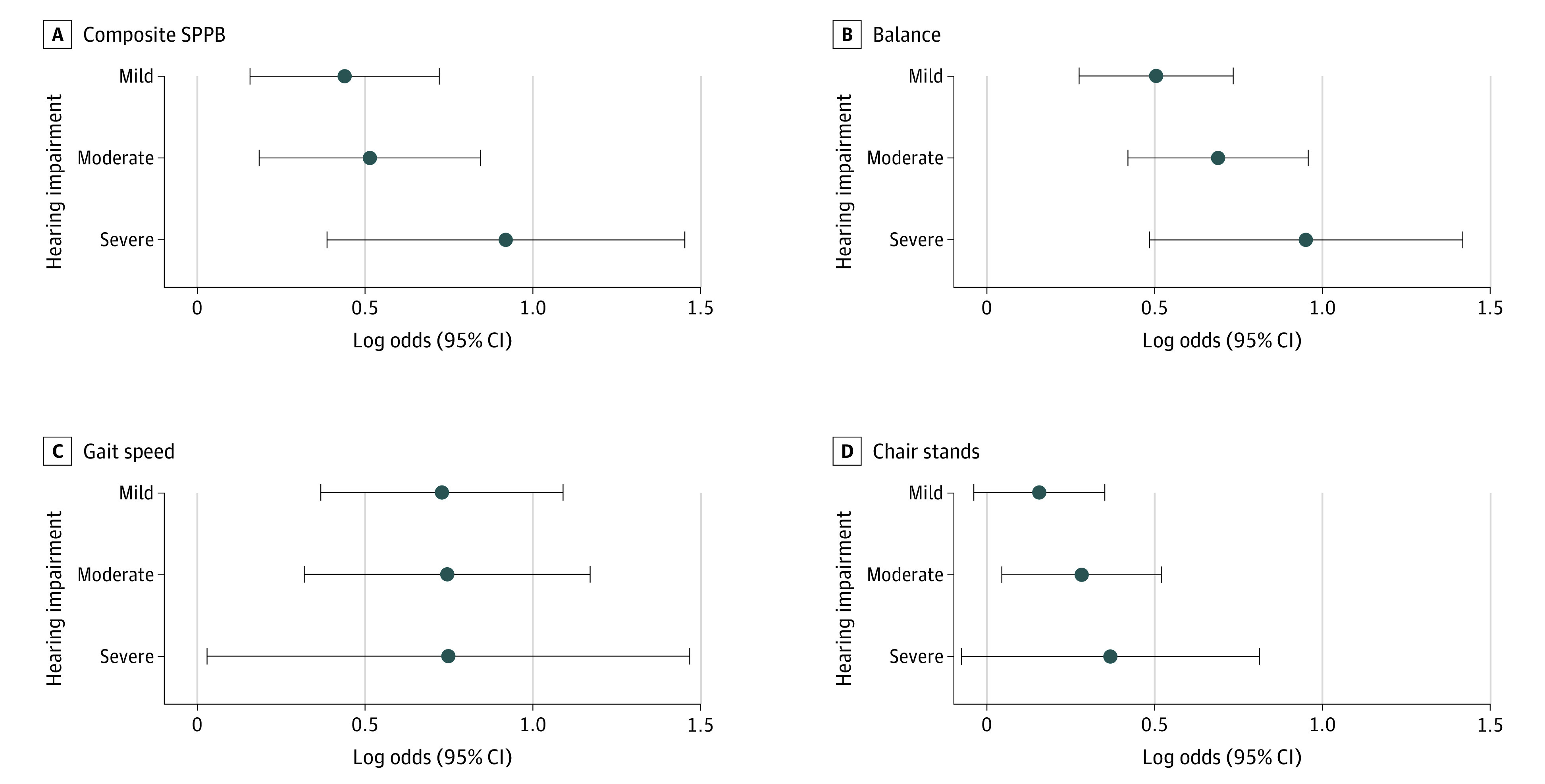
Adjusted Associations Between Hearing Categories and Low Vs High Short Physical Performance Battery (SPPB) Composite and Component Scores Markers indicate log odds compared with normal hearing; horizontal lines indicate 95% CIs. The model was adjusted for covariates in model 2: age, sex, race–center site, body mass index, educational level, occupational noise exposure, smoking status, and multimorbidity index.

When hearing was modeled categorically (Table 2 and Figure 1), in model 2 with normal hearing as the reference group, we found a graded (dose-response) and statistically significant association between hearing impairment categories and lower SPPB scores (severe hearing impairment vs normal hearing: β, −0.82; 95% CI, −1.30 to −0.34). There were also significant associations between hearing impairment categories and slower gait speed (severe hearing impairment vs normal hearing: β, −0.05 m/s; 95% CI, −0.09 to −0.02 m/s) and greater odds of having low SPPB composite (severe hearing impairment vs normal hearing: OR, 2.72; 95% CI, 1.60-4.63), low balance (severe hearing impairment vs normal hearing: OR, 2.72; 95% CI, 1.71-4.32), and low gait speed (severe hearing impairment vs normal hearing: OR, 2.16; 95% CI, 1.05-4.41) scores. There was no association between hearing impairment categories and chair stand test results (severe hearing impairment vs normal hearing: OR, 1.49; 95% CI, 0.96-2.32).

#### Walking Endurance

In model 2 (Table 2), during the TMW test, each 10-dB HL increase in BPTA was cross-sectionally associated with a shorter distance walked (β, –0.98 m; 95% CI, –1.69 to –0.26 m). Compared with participants with normal hearing, the mean distance walked among those with hearing impairment was shorter: mild, –2.10 m (95% CI, −4.24 to –0.04 m); moderate, –2.81 m (95% CI, –5.45 to –0.17 m); and severe, –5.31 m (95% CI, –10.20 to –0.36 m).

### Longitudinal Analysis Findings

**Figure 2.**
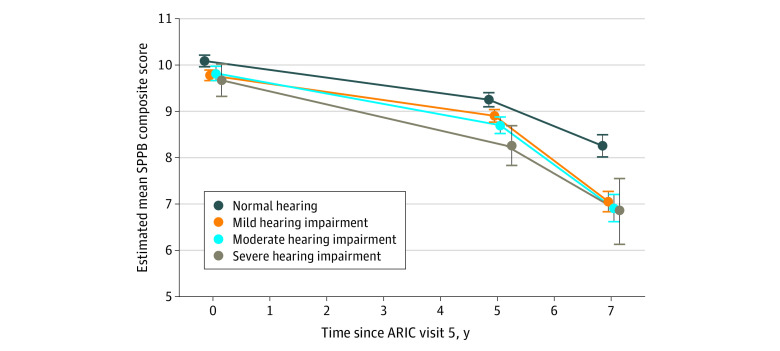
Estimated Mean Short Physical Performance Battery (SPPB) Composite Score Over Time Across Hearing Categories Adjusted for covariates in model 2: age, sex, race–center site, body mass index, educational level, occupational noise exposure, smoking status, and multimorbidity index. Error bars indicate 95% CIs. ARIC indicates Atherosclerosis Risk in Communities.

Participants with worse hearing demonstrated faster declines in SPPB composite scores over time (2-3 visits, with a maximum follow-up of 8.9 years; loss to follow-up = 600 participants [20%]) (Table 3 and Figure 2); interaction terms were as follows: BPTA × time 1 (first 5 years: difference, –0.002 [95% CI, –0.003 to –0.001]; *P* = .002) and BPTA × time 2 (year 5 onward: difference, –0.008 [95% CI, –0.012 to –0.003]; *P* = .001). Per 10-dB HL higher BPTA, the annual change in SPPB was −0.002 point/y (95% CI, −0.003 to −0.001 point/y) faster during the first 5 years and −0.008 point/y (95% CI, −0.012 to −0.003 point/y) faster from year 5 onward. Moreover, during the first 5 years, the rate of decline for those with normal hearing was −0.16 point/y (95% CI, −0.20 to −0.14 points/y); in comparison, those with moderate and severe hearing impairment had significantly faster rates of decline (rates of decline for hearing impairment: moderate, –0.22 point/y [95% CI, –0.26 to –0.19 point/y]; severe, –0.28 point/y [95% CI, –0.37 to –0.19 point/y]; Table 3 and Figure 2). From year 5 onward, the rate of decline for those with normal hearing accelerated to −0.50 point/y (95% CI, −0.61 to −0.39 point/y); in comparison, those with mild and moderate hearing impairment had significantly faster rates of decline (mild: –0.92 point/y [95% CI, –1.03 to –0.81 point/y]; moderate: –0.84 point/y [95% CI, –0.98 to –0.69 point/y]). In the secondary analysis (eTable 2 in the Supplement), the differences in physical function and walking endurance between hearing aid users and nonusers were not significant.

**Table 3.** Association Between Hearing Categories and Change in SPPB Composite Score

Hearing category	Year 0 to year 5				Year 5 to end of follow-up		
	Estimated annual rate of change in SPPB score, point/y (95% CI)^a^	Difference in the annual rate of change	*P* value	Estimated annual rate of change in SPPB score, point/y (95% CI)^a^		Difference in the annual rate of change	*P* value
Normal hearing (n = 934)	−0.16 (−0.20 to −0.14)	1 [Reference]	NA	−0.50 (−0.61 to −0.39)		1 [Reference]	NA
Mild HI (n = 1132)	−0.17 (−0.20 to −0.14)	−0.01 (−0.05 to 0.03)	.73	−0.92 (−1.03 to −0.81)		−0.42 (−0.58 to −0.26)	<.001
Moderate HI (n = 673)	−0.22 (−0.26 to −0.19)	−0.06 (−0.10 to −0.01)	.02	−0.84 (−0.98 to −0.69)		−0.34 (−0.52 to −0.16)	<.001
Severe HI (n = 116)	−0.28 (−0.37 to −0.19)	−0.11 (−0.21 to −0.02)	.02	−0.59 (−0.94 to −0.25)		−0.10 (−0.46 to 0.26)	.59

Abbreviations: HI, hearing impairment; SPPB, Short Physical Performance Battery.

^a^ Adjusted for age, sex, race–center site, body mass index, educational level, occupational noise exposure, smoking status, and multimorbidity index.

### Sensitivity Analyses

In ordinal logistic regression models, the odds of being in the next lower SPPB score were higher with worse hearing for all outcomes, but the OR was not statistically significant for the chair stand test (eTable 3 in the Supplement). No association between grip strength and hearing measures was found. The models that used the multimorbidity index with dementia yielded similar estimates and the same inferences as the index without dementia. The only exception was for the results for the association between severe hearing impairment and the gait speed binary outcome. Using the alternative multimorbidity index attenuated the estimate from an OR of 2.11 (95% CI, 1.02-4.33) to 1.97 (95% CI, 0.95-4.10).

## Discussion

In this cohort study of community-dwelling older adults in the US, hearing impairment was associated with poorer physical function and walking endurance in cross-sectional analysis and faster declines in physical function in longitudinal analysis. These associations were graded in general, with stronger associations among individuals with worse hearing. The differences in gait speed and walking endurance between participants with severe hearing impairment vs those with normal hearing (−0.05 and −5.3 m) were clinically meaningful according to previous literature.^22,23^ Collectively, these findings suggest that individuals with hearing impairment may be at greater risk for physical function limitations.

The association between hearing impairment and physical function has been studied previously. In a previous study, Deal et al^24^ found an association between hearing impairment and poorer physical function (SPPB scores) in a subsample of 250 ARIC participants. In that analysis, there were no associations of hearing impairment with poorer balance and slower gait speed, which might be explained by the smaller sample size. Chen et al^12^ found associations between hearing impairment and a faster decline in physical function over time in the Health Aging and Body Composition Study. Our findings are consistent with and expand on findings from this prior work. We studied each SPPB component separately, identifying stronger associations between hearing and balance and gait speed than between hearing and chair stands (Figure 1). Because of the increased complexity of balance and gait speed, integration of multiple inputs, and coordination of movement, it is likely that these functions rely more heavily on acoustic input from the environment compared with chair stands, which depend more on strength.^8,25^ In sensitivity analyses, we found no association between hearing and grip strength, supporting this assumption.

Mikkola et al^10^ investigated the associations between self-reported hearing and the SPPB components and did not find any associations after adjustment for confounders. This may be explained by the use of self-reported hearing, which has been shown to underestimate associations compared with objective measures of hearing function.^26^

We found an association between hearing and reduced walking endurance. Tests of walking endurance, which correlate with cardiorespiratory fitness,^27^ have been shown to be earlier factors associated with mobility limitations and disability compared with self-reported measures, particularly among well-functioning adults.^2,15^ In this context, our findings suggest that older adults with hearing impairment may be at an increased risk for mobility limitations. The association between hearing and walking endurance has been documented in only a few studies. Valjean et al^16^ assessed the association between audiometrically defined hearing impairment and reduced walking endurance in a 6-minute walk test. If the difference that we found in the TMW pace for participants with severe hearing impairment were maintained for 6 minutes, it would yield a difference (approximately 16 m) similar to that (16 m) reported by Valjean et al.^16^ However, their study was limited to Finnish women, and the findings were not statistically significant.

Multiple mechanisms could explain the associations found in the present study. First, a common cause (ie, cardiovascular disease) of hearing and physical impairment^28,29^could contribute to our findings. However, we adjusted for cardiovascular and sociodemographic risk factors, and our estimates were only slightly attenuated. Second, the inner ear hosts both the vestibular and auditory systems. Thus, damage to the inner ear could cause dysfunction in both systems, leading to an association between poorer hearing and balance. Although previous findings suggest that each sensory system has independent risk factors,^30^ we cannot rule out this mechanism because we were unable to adjust for vestibular function. Third, the association between hearing and physical function may be mediated through reduced cognitive resources,^31,32^ depression, social isolation, and reduced life space,^33,34,35,36^ resulting in less physical activity.^11,37,38^ These mechanisms would be particularly relevant for our findings related to walking endurance. In sensitivity analyses, we observed that when dementia was included in our adjustment variables (part of the multimorbidity index), it attenuated the association between severe hearing impairment and low gait speed scores. This finding is supportive of our theory that cognition is a mediator of the association between hearing and physical function. Fourth, hearing impairment may be associated with reduced physical function via reduced perception of auditory cues from the environment, which contribute to balance.^8,25^

We found no differences in physical function or walking endurance in association with hearing aid use (eTable 2 and eTable 4 in the Supplement). Participants who used hearing aids had higher socioeconomic status (associated with better physical function) but worse hearing, and they were older (associated with poorer function). Our measure of hearing aid use was limited to self-reports of yes or no, which did not characterize use patterns. These factors may explain the consistently null findings in the literature.^12^ Randomized clinical trials to elucidate the effect of hearing aid use on physical function are warranted.^39^

### Strengths and Limitations

This study has strengths. The study included a biracial population, 4 US sites, assessment of hearing using pure tone audiometry (clinical standard), performance-based tests of physical function and walking endurance, and repeated measures of physical function over 8 years. Our findings were robust to the adjustment for confounders and consistent across different statistical approaches (binary vs ordinal logistic regression from the sensitivity analysis).

This study also has limitations. First, the hearing assessment was conducted at visit 6; thus, in longitudinal analyses, we included measures of physical function that preceded the assessment of hearing. However, age-related hearing impairment progresses slowly at a rate of 1 to 2 dB HL per year^40,41^; thus, hearing at visit 5 would be correlated with hearing at visit 6. Also, the physical function at visit 5 would be unlikely to affect hearing acuity at visit 6. Moreover, we observed a significant decline in physical function between visit 6 and visit 7 in our models for participants with mild and moderate hearing impairment (Figure 2). Second, residual confounding by unmeasured variables, including subclinical cardiovascular disease and vestibular function, was possible. Third, 600 (20%) participants were lost to follow-up between visit 6 and visit 7. However, participants with worse hearing, who likely experienced faster declines in physical function, were more likely to be censored (eTable 5 in the Supplement). Under these conditions, if selection bias were present, it would likely be conservative.

Only 121 participants had severe hearing impairment. The relatively small sample size of this group could have reduced our statistical power to detect differences in physical functioning between participants with normal hearing and severe hearing impairment. However, we detected a significant association across multiple outcomes except in sensitivity analyses, in which the odds of having a low gait speed score were higher for participants with severe impairment. Although the study population included some Black participants, the sample was predominantly White. The lack of other racial/ethnic groups limits the generalizability of our findings. Further research in cohorts that include larger numbers of individuals of other racial/ethnic groups is warranted.

## Conclusions

In this cohort study, hearing impairment was associated with poorer physical function, reduced walking endurance, and faster decline in physical function over time. Because hearing impairment is amenable to prevention and management, it potentially serves as a target for interventions to slow physical decline with aging.

## Notes

Supplement. **eTable 1.** Scoring Cutoffs for the SPPB Scores **eTable 2.** Differences in Physical Function and Walking Endurance by Hearing Aid Use **eTable 3.** Sensitivity Analyses: Ordinal Logistic Regressions **eTable 4.** Demographic and Medical Characteristics at ARIC Visit 6 Comparing Hearing Aid Users Vs Nonusers **eTable 5.** Demographic and Medical Characteristics at ARIC Visit 6 Comparing Participants With Complete Follow-up Vs Dropout From Visit 6-7 **eFigure.** Study Design for Longitudinal Analysis
