# Supplementary material for: Association of Age-Related Hearing Impairment With Physical Functioning Among Community-Dwelling Older Adults in the US
Source: JAMA Netw Open. 2021 Jun 25;4(6):e2113742. doi: 10.1001/jamanetworkopen.2021.13742 (PMC8233700; doi:10.1001/jamanetworkopen.2021.13742)
Supplement: Supplement. — eTable 1. Scoring Cutoffs for the SPPB Scores eTable 2. Differences in Physical Function and Walking Endurance by Hearing Aid Use eTable 3. Sensitivity Analyses: Ordinal Logistic Regressions eTable 4. Demographic and Medical Characteristics at ARIC Visit 6 Comparing Hearing Aid Users Vs Nonusers eTable 5. Demographic and Medical Characteristics at ARIC Visit 6 Comparing Participants With Complete Follow-up Vs Dropout From Visit 6-7 eFigure. Study Design for Longitudinal Analysis [file jamanetwopen-e2113742-s001.pdf]

Supplementary Online Content

Martinez-Amezcua P, Powell D, Kuo PL, et al. Association of age-related hearing impairment with physical functioning among community-dwelling older adults in the US. *JAMA Netw Open*. 2021;4(6):e2113742.
doi:10.1001/jamanetworkopen.2021.13742

eTable 1. Scoring Cutoffs for the SPPB Scores
eTable 2. Differences in Physical Function and Walking Endurance by Hearing Aid Use
eTable 3. Sensitivity Analyses: Ordinal Logistic Regressions
eTable 4. Demographic and Medical Characteristics at ARIC Visit 6 Comparing Hearing Aid Users Vs Nonusers
eTable 5. Demographic and Medical Characteristics at ARIC Visit 6 Comparing Participants With Complete Follow-up Vs Dropout From Visit 6-7
eFigure. Study Design for Longitudinal Analysis

This supplementary material has been provided by the authors to give readers additional information about their work.

© 2021 Martinez-Amezcua P et al. *JAMA Network Open*.

eTable 1. Scoring Cutoffs for the SPPB Scores

Physical function category	*Total SPPB Score*	*Binary score*
Poor	0-6	Low
Intermediate	7-9	High
Good	10-12	
Standing Positions	*Balance Score*	*Binary score*
Unable to hold side-by-side	0	Low
Side-by-side for 10 seconds	1	
Semi-tandem for10 seconds	2	
Tandem >3, but <10 seconds	3	High
Tandem for 10 seconds	4	
Usual gait (m/s) over 4-meters	*Gait Speed Score*	*Binary score*
Unable to complete the walk	0	Low
≤ 0.46	1	
>0.46 and ≤0.64	2	
>0.64 and ≤0.83	3	High
>0.83	4	
Time to complete 5 stands (seconds)	*Chair Stands Score*	*Binary score*
Unable to complete the stands	0	Low
16.7 to 60	1	
13.7 to <16.7	2	
11.2 to <13.7	3	High
<11.2	4	

Abbreviations: SPPB, Short Physical Performance Battery

© 2021 Martinez-Amezcua P et al. *JAMA Network Open*.

**eTable 2. Differences in Physical Function and Walking Endurance by Hearing Aid Use.**		
Outcomes	No Hearing Aid Use (Reference)	Hearing Aid Use
	OR (95% confidence interval) for low SPPB scores‡, binary logistic regression	
Composite SPPB score	1	0.85 (0.57 to 1.29)
Balance Score	1	0.90 (0.63 to 1.28)
Gait Speed Score	1	0.84 (0.48 to 1.48)
Chair Stand Score	1	1.11 (0.79 to 1.56)
	β coefficient (95% confidence interval)	
SPPB composite score, points	0	0.27 (-0.11 to 0.66)
Distance in the TMW, meters	0	3.07 (-0.79 to 6.93)
Gait speed, m/s	0	0.02 (-0.01 to 0.05)
Time to complete chair stands, seconds	0	0.14 (-0.54 to 0.82)

Abbreviations: SPPB, Short Physical Performance Battery; TMW, Two-minute Walk
‡: (≤6 for composite ≤2 for each component)
Adjusted for: age, sex, race-center, BMI, education, occupational noise exposure, smoking, and multimorbidity index

© 2021 Martinez-Amezcua P et al. *JAMA Network Open*.

eTable 3. Sensitivity Analyses: Ordinal Logistic Regressions				
Outcome	Normal Hearing (Reference)	Mild Hearing Impairment	Moderate Hearing Impairment	Severe Hearing Impairment
Composite SPPB score	1	**1.39 (1.15 to 1.66)**	**1.49 (1.19 to 1.86)**	**1.95 (1.30 to 2.91)**
Balance Score	1	**1.48 (1.22 to 1.80)**	**1.67 (1.33 to 2.11)**	**2.14 (1.44 to 3.18)**
Gait Speed Score	1	**1.39 (1.12 to 1.72)**	**1.57 (1.21 to 2.03)**	**1.95 (1.26 to 3.00)**
Chair Stand Score	1	1.06 (0.91 to 1.25)	1.17 (0.97 to 1.42)	1.27 (0.88 to 1.82)

Abbreviations: SPPB, Short Physical Performance Battery; TMW, Two-minute Walk
‡: (≤6 for composite ≤2 for each component)
OR: Represent odds ratios of being in the next lower category (for composite score: high, intermediate, poor; for SPPB components, next lower score (0-4)
Adjusted for: age, sex, race-center, BMI, education, occupational noise exposure, smoking, and multimorbidity index

© 2021 Martinez-Amezcua P et al. *JAMA Network Open*.

**eTable 4. Demographic and medical characteristics at ARIC visit 6 comparing hearing aid users vs. nonusers**				
	**No. (%)**			
**Characteristics**	***Total (n=811)***	***Nonusers (n=337)***	***Hearing Aid users (n=474)***	***p-value***
Age, mean (SD), years	81.2 (4.9)	80.8 (4.9)	81.5 (4.9)	0.034
Men	460 (56.7)	165 (49.0)	295 (62.2)	<0.001
White race	735 (90.6)	282 (83.7)	453 (95.6)	<0.001
Body Mass Index, mean (SD), kg/m^2	27.9 (5.0)	28.2 (5.5)	27.7 (4.6)	0.20
Multimorbidity Index, number of conditions				0.078
0	370 (51.0)	163 (54.7)	207 (48.5)	
1-2	293 (40.4)	106 (35.6)	187 (43.8)	
≥3	62 (8.6)	29 (9.7)	33 (7.7)	
Education				0.006
Less than high school	108 (13.3)	54 (16.0)	54 (11.4)	
High school or equivalent	373 (46.0)	167 (49.6)	206 (43.5)	
More than high school	330 (40.7)	116 (34.4)	214 (45.1)	
Occupational noise exposure	277 (34.2)	104 (30.9)	173 (36.5)	0.095
Smoking Status				0.013
Never	316 (39.0)	144 (42.7)	172 (36.3)	
Former	434 (53.5)	161 (47.8)	273 (57.6)	
Current	61 (7.5)	32 (9.5)	29 (6.1)	
SPPB Composite, median (IQI), score	9.0 (7.0-11.0)	9.0 (6.0-11.0)	9.0 (7.0-11.0)	0.005
Low SPPB composite score	183 (22.6)	87 (25.8)	96 (20.3)	0.062
Low SPPB balance score	308 (38.0)	136 (40.4)	172 (36.3)	0.24
Low SPPB gait score	90 (11.1)	50 (14.8)	40 (8.4)	0.004
Low SPPB chair stand	494 (60.9)	215 (63.8)	279 (58.9)	0.16
Gait speed, mean (SD), m/s	0.91 (0.22)	0.88 (0.23)	0.93 (0.20)	0.001
Time to complete stands, mean (SD), seconds	14.8 (4.6)	15.1 (4.7)	14.6 (4.6)	0.18
TMW, mean (SD), meters	135.6 (28.7)	132.2 (30.6)	137.9 (27.2)	0.012
BPTA, mean (SD), dB	51.7 (9.4)	48.2 (6.9)	54.2 (10.2)	<0.001

Abbreviations: HI: Hearing impairment; SPPB, Short Physical Performance Battery; IQI, Interquartile interval (25th percentile-75th percentile); TMW, Two-minute walk; BPTA, Better hearing ear's pure-tone average.
p-values were determined using chi-square for categorical variables and ANOVA for continuous variables.

© 2021 Martinez-Amezcua P et al. *JAMA Network Open*.

**eTable 5. Demographic and medical characteristics at ARIC visit 6 comparing participants with complete follow-up vs. dropout from visit 6-7.**				
	**No. (%)**			
**Characteristics**	***Total (n=2,956)***	***Complete follow-up (n=2,356)***	***Dropout (n=600)***	***p-value***
Age, mean (SD), years	79.1 (4.6)	78.9 (4.5)	80.0 (4.9)	<0.001
Men	1,234 (41.7)	967 (41.0)	267 (44.5)	0.13
White race	2,356 (79.7)	1,850 (78.5)	506 (84.3)	0.002
Body Mass Index, mean (SD), kg/m^2	28.3 (5.3)	28.4 (5.3)	27.9 (5.3)	0.038
Multimorbidity Index, number of conditions				0.002
0	1,461 (49.4)	1,202 (51.0)	259 (43.2)	
1-2	1,126 (38.1)	875 (37.1)	251 (41.8)	
≥3	369 (12.5)	279 (11.8)	90 (15.0)	
Education				0.049
Less than high school	316 (10.7)	242 (10.3)	74 (12.3)	
High school or equivalent	1,223 (41.4)	959 (40.7)	264 (44.0)	
More than high school	1,417 (47.9)	1,155 (49.0)	262 (43.7)	
Occupational noise exposure	712 (24.1)	551 (23.4)	161 (26.8)	0.078
Smoking Status				0.42
Never	1,164 (39.4)	940 (39.9)	224 (37.3)	
Former	1,580 (53.5)	1,245 (52.8)	335 (55.8)	
Current	212 (7.2)	171 (7.3)	41 (6.8)	
SPPB Composite, median (IQI), score	9.0 (8.0-11.0)	10.0 (8.0-11.0)	9.0 (7.0-11.0)	<0.001
Low SPPB composite score	520 (17.6)	386 (16.4)	134 (22.3)	<0.001
Low SPPB balance score	836 (28.3)	640 (27.2)	196 (32.7)	0.008
Low SPPB gait score	276 (9.3)	197 (8.4)	79 (13.2)	<0.001
Low SPPB chair stand	1,698 (57.4)	1,322 (56.1)	376 (62.7)	0.004
Gait speed, mean (SD), m/s	00.94 (0.22)	0.95 (0.21)	0.90 (0.23)	<0.001
Time to complete stands, mean (SD), seconds	4.6 (14.6)	14.4 (4.6)	15.0 (4.8)	0.014
TMW, mean (SD), meters	137.7 28.4	139.1 (27.9)	131.9 (29.6)	<0.001
Hearing categories				<0.001
Normal	973 (32.9)	811 (34.4)	162 (27.0)	
Mild impairment	1,170 (39.6)	924 (39.2)	246 (41.0)	
Moderate impairment	692 (23.4)	537 (22.8)	155 (25.8)	
Severe impairment	121 (4.1)	84 (3.6)	37 (6.2)	

© 2021 Martinez-Amezcua P et al. *JAMA Network Open*.

Abbreviations: HI: Hearing impairment; SPPB, Short Physical Performance Battery; IQI, Interquartile interval (25th percentile-75th percentile); TMW, Two-minute walk; BPTA, Better hearing ear's pure-tone average.
p-values were determined using chi-square for categorical variables and ANOVA for continuous variables.

eFigure 1. Study Design for Longitudinal Analysis

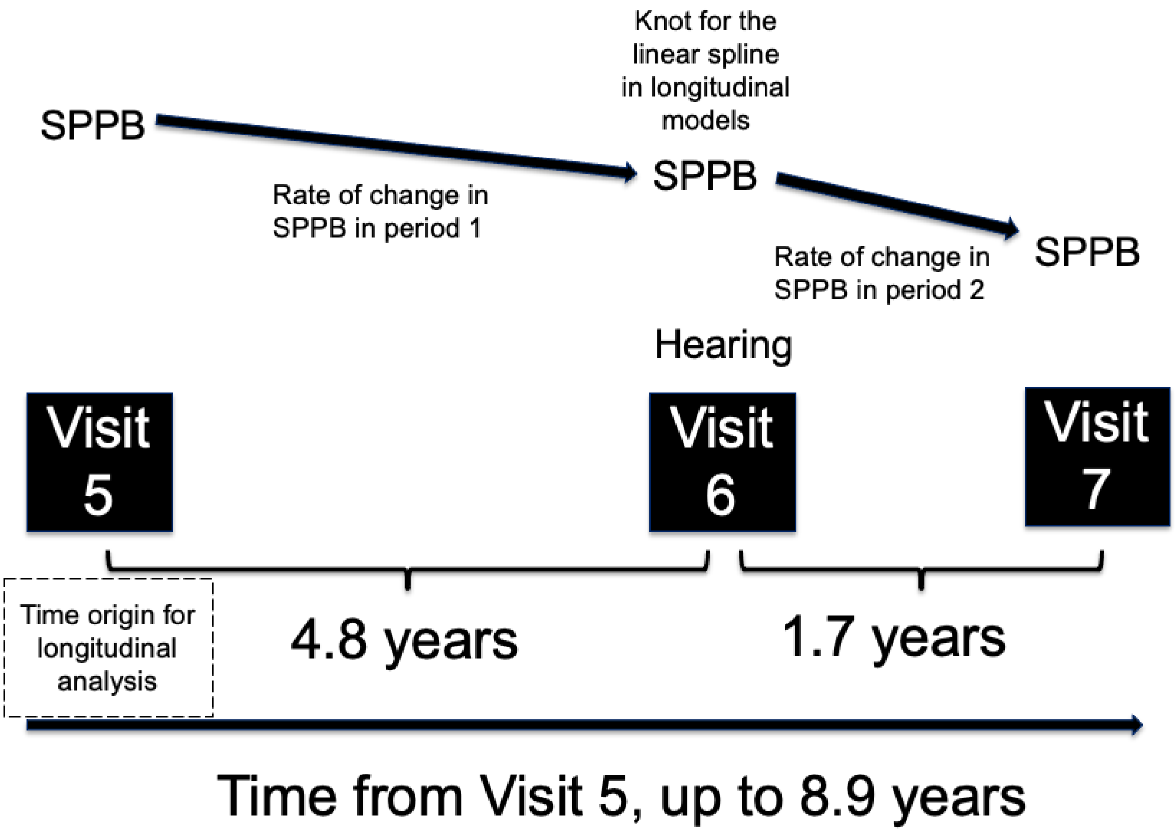

© 2021 Martinez-Amezcua P et al. *JAMA Network Open*.
